# Genomic Education at Scale: The Benefits of Massive Open Online Courses for the Healthcare Workforce

**DOI:** 10.3389/fgene.2019.01094

**Published:** 2019-11-13

**Authors:** Michelle Bishop, Edward Miller, Amelia McPherson, Siobhan Simpson, Stuart Sutherland, Anneke Seller

**Affiliations:** ^1^Genomics Education Programme, Health Education England, Birmingham, United Kingdom; ^2 ^National School of Healthcare Science, Health Education England, Birmingham, United Kingdom

**Keywords:** workforce development, genomic medicine, Massive Open Online Course, evaluation, genomic education, multi-disciplinary education, online learning

## Abstract

To support the delivery of the UK’s 100,000 Genomes Project, Health Education England’s Genomics Education Programme developed a suite of resources, including a 3-week Massive Open Online Course (MOOC) on whole genome sequencing *via* the FutureLearn platform. This MOOC is a synchronous learning event, with course educators and mentors (NHS healthcare science trainees in genomics) facilitating the experience in real time. Crucially, the platform allows participants to interact and learn from each other’s experiences. The evaluation of the course was considered from the learners’ and mentors’ perspectives. Perceptions of course relevance were examined through analysis of learner comments made throughout the course and responses to an end-of-course survey. Evaluation of mentors’ experiences focused on how prepared they felt to undertake their role and the value and benefit of their experience. Data was collected through a mixed methods study after the first two runs of the course. Here we present findings from 440 learners who provided end-of-course reflections, 360 learners who completed the post-course survey and 14 mentors who facilitated the course. The course met learners’ needs by providing a greater understanding of whole genome sequencing and the application of this technology in healthcare. Learners also highly valued the engagement with mentors. Mentors appreciated the experience and identified areas of professional development gained through the mentoring experience. Our findings show that a team of specialist healthcare course mentors engaging with a range of different healthcare professional MOOC learners in online conversation can enhance the learners’ experiences and provide a beneficial continuing professional development opportunity for mentors.

## Introduction

With the establishment of genomic medicine initiatives around the world the use of genomic information is increasingly being used as part of routine clinical practice (Stark et al., 2019). There are many challenges to successfully integrating genomics into healthcare systems, one of which is workforce capacity and capability (Manolio et al., 2015). In establishing the 100,000 Genomes Project, England became one of the first countries to introduce whole genome sequencing (WGS) into an established health system (NHS England 2019b). Alongside the scientific and clinical discoveries, this multifaceted project provided a unique opportunity to implement a co-ordinated approach to workforce education and development. Health Education England’s Genomics Education Programme (GEP) was established to provide the educational support to staff delivering the project (www.genomicseducation.hee.nhs.uk).

To prioritize the education and training needs of the workforce, the GEP and key stakeholders undertook an exercise to identify the resources required to support the clinical and scientific activities across the 100,000 Genomes Project pipeline. While most of the resources supported areas specific to the project protocol, others had wider clinical applicability. One of these was a Massive Open Online Course (MOOC) ’Whole Genome Sequencing: Decoding the Language of Life and Health’ (https://www.futurelearn.com/courses/whole-genome-sequencing).

MOOCs are defined as open access courses for unlimited numbers of learners (Yousef et al., 2014). While MOOCs have been in existence for over a decade, the modern MOOC movement, characterized by the development of dedicated platforms and providers delivering online courses to large numbers of learners, began in 2012 (Pappano, 2012). MOOCs offer open access learning irrespective of geographical, professional or educational settings compared to other types of online learning (see **Table 1**). By 2018 the total number of learners signed up to at least one MOOC had surpassed 100 million (Shah, 2018). MOOCs with a healthcare focus have seen rapid growth internationally in both the number of courses available and the number of registered learners (Liyanagunawardena and Williams 2014, Shah, 2018). MOOCs have enabled healthcare professionals to learn at scale and pace across professional and geographical boundaries (Wewer Albrechtsen et al., 2017, Liyanagunawardena and Aboshady 2018, Sneddon et al., 2018).

**Table 1:** High level comparison of FutureLearn MOOCs with other forms of large-scale professional online learning.

	Future Learn MOOCs	Other Types of Large Scale Professional Online Learning
Access	Open to anyone who has internet access. Free to join with optional upgrades for a fee.	Access can often be through a learning management system or *via* a subscription model, which may restrict access to certain professional groups or fee-paying learners.
Type of Learning Event	Synchronous. Courses have specified start dates, so learners can move through the course in a cohort. Courses run for weeks, with on average 2/3 hours of learning per week.	Often asynchronous. Learners can register and undertake courses and consume resources at any time. Courses range in length.
Facilitation	Facilitation is a key component of the FutureLearn model and can be done by the course authorship team, dedicated mentors and indeed other learners from within the learner community.	Standalone courses and resources for learners to work through independently – without facilitation – is the more common professional online learning model.
Types of Learners	Learner cohort is highly heterogeneous, due to the open nature of the platform	Learners are more likely to be from the same professional group.
Credit/Qualification Bearing	May have accreditation with professional bodies for CPD points, or form part of an accredited university module.	May have accreditation with professional bodies for CPD points, or form part of an accredited university module.

Another area of rapid evolution is the accreditation/credentialing of MOOCs. As MOOC providers have increasingly focused on supporting professional development, they have developed an array of paid-for offerings on top of free, open courses so that learners can earn certificates of completion, credentials, professional body CPD points, and academic credit (Brown, 2018).

The GEP chose to partner with FutureLearn (www.futurelearn.com), a UK-based MOOC platform launched in 2013 that, at the time of writing had 18 courses that focused on genomics from a healthcare perspective. FutureLearn courses are delivered synchronously, with specific start and end dates for each run. Courses are structured into weeks, with each week containing a number of ’steps’. The content in each step can be delivered *via* different formats, the most common being text and video. Additionally, courses can contain test steps (for formative and summative testing) and poll steps for learners to vote on key topics. A defining feature of the FutureLearn platform is that learners can comment throughout the course and ’like’ and comment on each other’s comments. Course designers can also include specific discussion steps, actively encouraging reflection and communication amongst learners. The platform also allows course providers to allocate the role of mentor to specific members of the course delivery team who are tasked with supporting and responding to learners.

Mentors have an essential role to play throughout a MOOC run, fostering a social and connected learning experience (Leon Urrutia et al., 2015). As learners come from very diverse backgrounds, mentors can support the course delivery team in handling the variety and quantity of comments and questions raised. Furthermore, mentors act as mediators to facilitate learning and encourage learners’ engagement with the course (Kop, 2011, Watolla, 2016). The open accessibility of MOOCs mean they can suffer from learner attrition with data from one of the biggest providers, edX, showing 52% of registered learners never start the course (Reich and Ruipérez-Valiente, 2019). Some commentators suggest the inability to facilitate and support such a varied cohort of learners may be one explanation for the loss of engagement (Hone and El Said, 2016).

This paper summarizes the process the GEP followed to develop the MOOC, including the recruitment and training of mentors. It reports on the short-term outcomes from the evaluation plan and comments on how MOOCs could be used to support healthcare workers’ ongoing professional development.

## Methods

### Development of the Course

**Figure 1:**
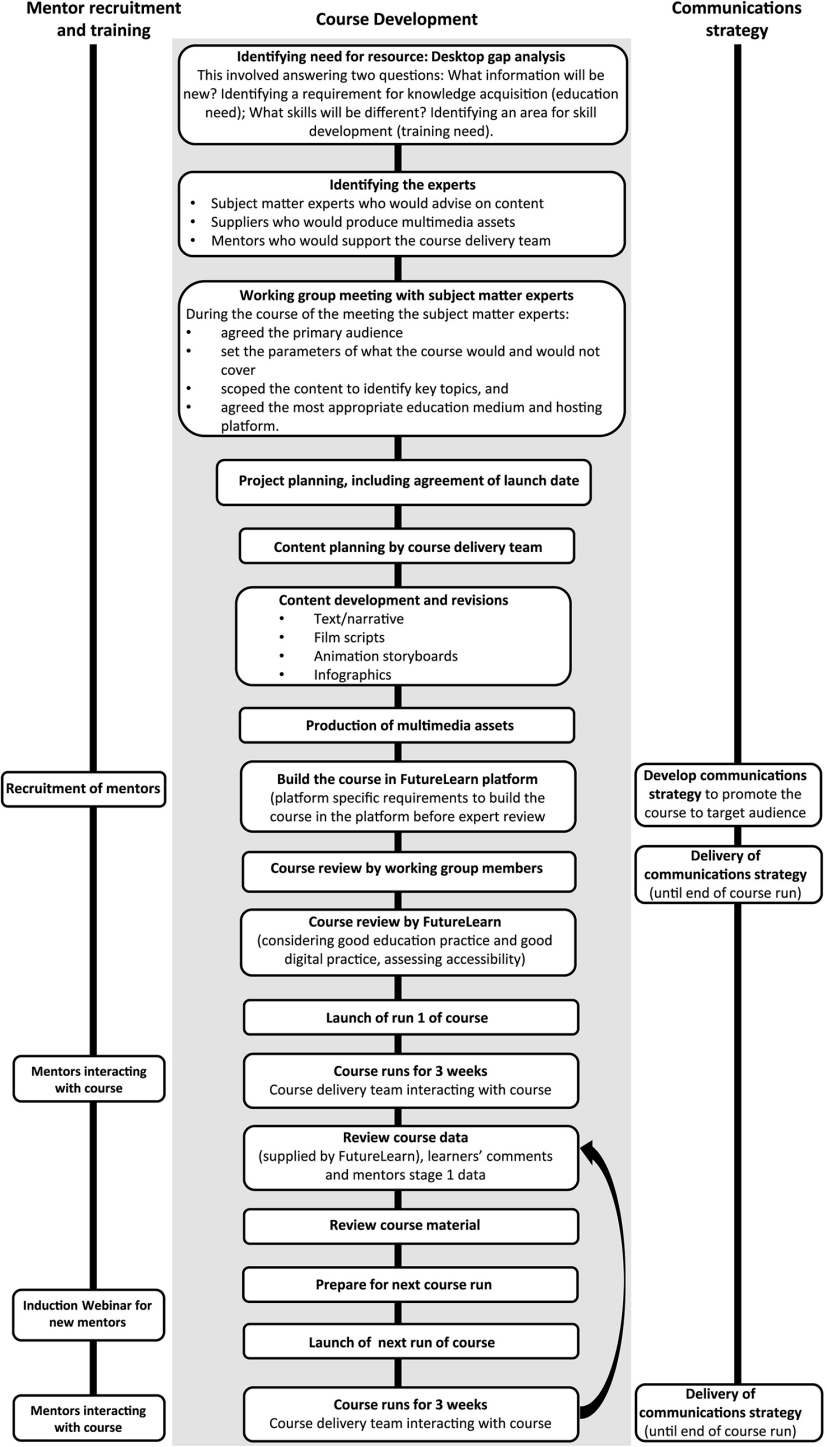
Stages of developing the MOOC ’Whole Genome Sequencing: Decoding the language of life and health’.

**Figure 1** outlines the steps taken to develop the MOOC, including the recruitment and training of mentors and the communication strategy. The aim of this MOOC was to increase understanding of WGS technology and its application in healthcare, to a broad range of NHS professionals who had limited understanding and/or exposure to genomic testing. The development of the course was overseen by a course delivery team which included the authors.

### Recruitment and Training of Mentors

**Figure 2:**
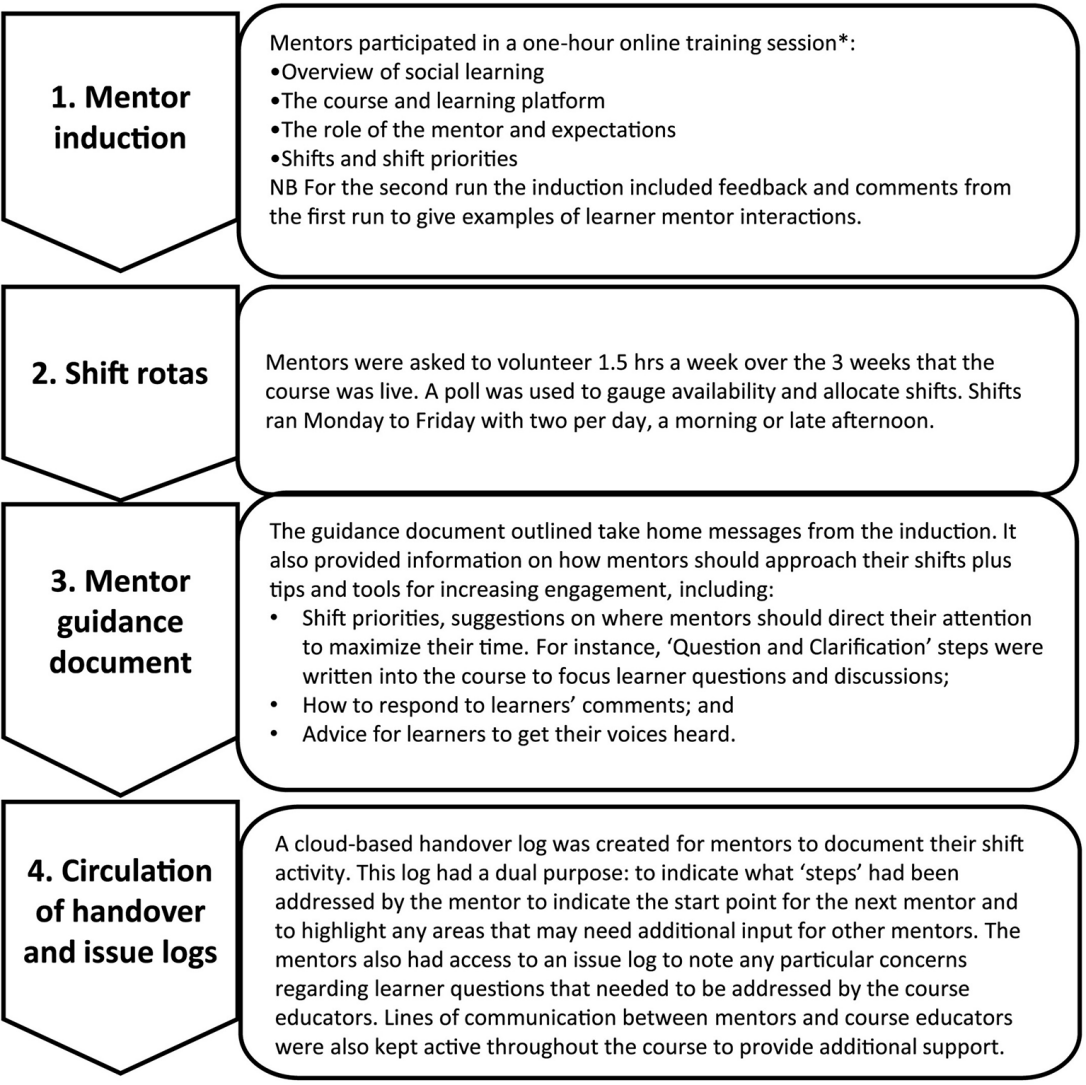
Model to promote a connected mentoring team based on the mentor model described by Leon Urrutia et al. (2015). *Covering the expected roles of online mentors as outlined by Berge (1995).

A decision was made by the course team to recruit healthcare professionals who are specialists in genomics to support the facilitation of the MOOC. The National School of Healthcare Science (www.nshcs.hee.nhs.uk/) invited individuals who were enrolled in NHS healthcare science training programs in genomics. These included individuals on the Higher Specialist Scientist Training (HSST) program, a five-year doctoral level program, and individuals in their final years of the Scientist Training Program (STP), a three-year master’s-level program. Recruitment was targeted to trainees rather than practising healthcare professionals as trainees could use this experience as evidence for competencies in their curriculum. In this paper we present the evaluation data of the mentor model from runs 1 and 2. Sixteen individuals (12 HSST trainees, three STP trainees, one course educator) were recruited to mentor runs 1 and 2, with five mentors involved in both runs. Prior to each run, new mentors participated in an induction webinar, and were provided with guidance documents to support their mentoring activities. Our mentor model (shown in **Figure 2**) was based on that described by Leon Urrutia et al. (2015), where university graduate students (Leon Urrutia et al., 2016) and faculty (Leon Urrutia et al., 2015) were used as mentors for non-healthcare related FutureLearn MOOCs.

### Evaluation of Learners’ Experiences and the Mentor Model

To evaluate learners’ experiences and identify any changes to learners’ knowledge, we analyzed two sources of secondary data provided by FutureLearn. To evaluate the effectiveness of the mentor model, we adopted a mixed methods approach to data collection and analysis: stage 1 following run 1, and stage 2 following run 2. This work has been categorized as service evaluation, and as such did not require NHS ethics approval. The GEP worked within the data governance framework of Health Education England.

#### Analysis of Learners’ Comments Posted During the Course

A step was created at the beginning of the course (Step 1.1) for learners to introduce themselves and outline what they hoped to gain from the course. At the end of the course (Step 3.17) learners were given the opportunity to reflect on what they had learnt and their overall experience. Comments from both steps from all six runs were downloaded from the FutureLearn platform, anonymised and uploaded to NVivo 10 for data management. Content analysis was used to categorize the comments from Step 1.1 into professional groups, then thematic analysis was undertaken using the constant comparison approach as first described by Glaser and Strauss (1967). Thematic analysis of the comments from Step 3.17 was also conducted using the constant comparison approach. Quotes presented in this paper are from all six runs and have been de-identified to remove any reference to the learner or their profession.

#### FutureLearn Post-Course Survey

At the end of each run FutureLearn administers a post-course survey. This paper presents findings from the post-course survey on learners’ views of the platform and how they rated the content of the course. All survey responses were anonymous. The authors requested the data from the post-course survey, and this was provided for runs 1 and 3–6. Data from the post-course survey for run 2 was not available for analysis. The questions included in the survey were decided by FutureLearn, and not all questions were present in each survey. Learner’s satisfaction with the FutureLearn platform, interactions with other learners and mentors, and course content (including complexity) was assessed through numerous statements, with learners asked to rate each statement from 1 (strongly liked/very satisfied) to 5 (strongly disliked/very dissatisfied). For this paper we use the term ’satisfied/very satisfied’ for simplicity in reporting. An additional question asking if learners worked for the NHS or Public Health England (Yes/No response) was also included in the post-course survey for runs 1, 3 and 4 at the authors’ request. Descriptive statistics were used to describe the findings.

#### Evaluation of the Effectiveness of the Mentor Model

Stage 1: A survey was administered to the mentors who facilitated run 1 *via* an anonymous questionnaire. Consent was implied by return of the questionnaire. Mentors were asked to provide their own description of their role and their activity as a mentor. Mentors’ preparedness and feeling supported in their role, their experience of being a mentor, and the challenges they faced, were assessed through a series of statements where respondents were asked to rate each statement from 1 (strongly disagreed) to 5 (strongly agreed). Throughout the questionnaire mentors were asked open-ended questions to comment or expand on their responses. Descriptive statistics were used to describe the findings. Thematic analysis of the solicited comments was undertaken using a constant comparison approach.

Stage 2: Recruitment letters were emailed to the mentors who facilitated run 2. Participants who expressed an interest contacted MB to schedule a telephone interview. Mentors who expressed an interest but were unable to attend an interview were sent a questionnaire *via* email. Verbal consent was obtained prior to beginning the phone interviews. For mentors who received the questionnaire, consent was implied by return of the completed questionnaire. A semi-structured interview guide was used in the interviews, which were approximately 20 min per participant. The interview guide was informed from the findings of stage 1 and explored mentors’ experiences, their perceptions of learners, and their impressions of the mentoring experience. The questionnaire was based on the topic guide and covered the same key areas. All interviews were recorded and transcribed verbatim. Thematic analysis of the interview transcripts and the responses to the questionnaire were conducted using a constant comparison approach. Analysis of the transcripts and questionnaire responses was undertaken by MB and checked by StS for consistency. NVivo 11 was used for data management.

## Results

The MOOC has run six times between September 2016 and October 2018. Over these runs 19,683 individuals have enrolled on the course, of which 45.2% have entered the course and viewed at least one step (n = 8,894). Of these learners 28.9% (n = 2,573) were also ’social learners’—defined as posting at least one comment on any step.

### Learners Come From Five Different Sectors

Analysis of the comments from Step 1.1 showed learners came from five different sectors (See **Table 2**). Each group had specific motivations for undertaking the course, with healthcare professionals primarily wanting to improve their knowledge of genomics and/or WGS and increase their awareness of the clinical utility of WGS. Data from post-course surveys showed that of the 316 people who completed the questionnaire (runs 1, 3 and 4), 32% worked within the NHS (n = 101).

**Table 2:** High level overview of learners and their motivations for participating in the MOOC*.

Sector	Sub-groups	Examples	Motivation
Healthcare	Specialists	Geneticists, Scientists, Genetic Counsellors	Refresh their knowledge Hear from patients and wider clinical workforce
	Wider clinical staff	Medical, Nursing, Healthcare Scientists, AHP, Public Health	To find out more as know relevant for future role Understand where genomics will impact on healthcare
	Non-clinical staff	Project Managers, Business Managers, Directors	To understand more about their clinical colleagues’ work
Academia	Academics/ Researchers	Bench researchers, lecturers, teachers	Consider the impact of genomics in the clinic
	Students	Final year(s) school through to PhD	Improve knowledge and understanding
Industry	Scientific staff	Researchers	Refresh knowledge To find out more as new to the area
	Non-scientific staff	Business Managers	To understand the science
Public	’Professional’ role	Lawyer, Author etc.	Professional and personal; interest
	Lay people		Personal interest
Patient	Personal history		Undergoing WGS Want to know more about technology
	Family history	Including parents	

*Please note that additional demographic information such as age and number of years of experience was not available for analysis.

### The Course Met Learners’ Needs and Provided a Strong Foundation in Genomic Knowledge

From the 440 comments analyzed from Step 3.17 the course appeared to meet the needs and expectations of learners. After completing the MOOC, learners stated that they had:

- an increased knowledge of the scientific and clinical aspects of WGS (including current limitations of WGS);
- a greater awareness of the ethical considerations of WGS;
- a wider appreciation of the application of genomics in healthcare; and
- an awareness of their own role in the WGS clinical pathway (where appropriate).

For most learners, the course was pitched at the right level. However, a small minority (n = 3) felt the course content and discussion was too simple with one commenting the content was presented using an “unscientific narrative” (Psychiatrist). These findings corresponded with the results from the post-course survey, where 343 of the 360 respondents (93.3%) stated they were satisfied or very satisfied with the course content. In runs 4 and 5 respondents were also asked to rate the level of complexity of the course, with 91.8% of the 98 respondents stating they were satisfied or very satisfied. For those who were not satisfied, it was either because the information was considered “very difficult” (n = 2) or “way too low” (n = 6).

### Healthcare Professionals Intended to Apply Their New Knowledge in Their Practice

As demonstrated by the comments from step 3.17, completing this course increased learners’ genomic knowledge and, as one learner commented, this “helped secure a lot of terms and processes by putting them in context” (Biomedical Scientist). Those who were healthcare professionals stated they would be more confident in engaging in conversations with colleagues and having informed discussions with patients. As well as increasing their knowledge, some learners mentioned that they would take away examples of how to explain genomic concepts in an understandable way as they found they had “the tools to explain this to others” (Pharmacist).

A minority of learners stated they would use this knowledge to evaluate the benefit of using WGS within their own specialism. While it is not clear which health setting these professionals worked in, the small number who identified as working within the NHS stated that following completion of the course, they looked forward to discussing how WGS could be applied in their area of practice.

### The FutureLearn Platform Enhanced Learners’ Experiences

Learners who commented on Step 3.17 were complementary of the FutureLearn platform. The three most common reasons related to: learning as a cohort, the ability to comment on each step, and the flexible nature of the course as they could complete their learning at a time and a pace that suited them.

Learners commented on the positive experience of learning as a cohort, with one likening it to “being in a class” (Biomedical Scientist). Other learners referred to each other within the comments as “class-mates” and “fellow learners”. Many of the 440 learners who provided their final reflections stated that they enjoyed reading the comments and looking at the diversity of views amongst the cohort, particularly those from patients and their families. One learner even stated that contributing to discussions was a great way to check their own understanding and to reflect on what was covered in the course.

These findings corresponded with the results from the post-course survey, where 77.6% of the respondents to the post-course surveys (n = 357) were satisfied or very satisfied with the discussions with other learners.

Learners also valued the mentors’ contributions to the course discussion and enjoyed engaging in dialogue with individuals who worked at the “coalface” of genomic medicine. Learners appreciated the disciplined nature in which mentors responded to learners’ questions which, according to some learners, “isn’t the case on all courses” (Secondary school teacher).

A question about interacting with the course team was only asked in the post-course survey for runs 3–5 (n = 129). However, 93.0% of these respondents were satisfied or very satisfied with their interactions with the course team and reading comments posted by the educators or mentors.

### Mentors’ Experience of Facilitating a MOOC

Nine out of the 11 mentors involved in the first run completed the stage 1 survey. All 10 mentors who facilitated the second run of the MOOC participated in stage 2 of the evaluation: six participated in a telephone interview and four responded to a structured questionnaire. The results from both stages 1 and 2 are presented together.

#### Mentors Felt Well Prepared and Enjoyed Facilitating the MOOC

All nine respondents of the stage 1 survey agreed or strongly agreed that the introductory webinar prepared them for the role. One respondent stated:

“I think everyone was a little nervous before doing it as it was quite new, but after having a go for half an hour or so it soon became clear and quite enjoyable” (Survey Responce)

In addition, all stage 1 respondents agreed or strongly agreed that the ongoing guidance and support from the GEP helped them perform their role and focus their activities during the MOOC run.

When asked to provide their own description of their role and activity as a mentor, the most common themes from the stage 1 survey were those of being helpful and supporting learners. In some cases, this involved answering learners’ questions; for others it involved signposting to additional resources and references. For some, responding to individual learners’ needs was the most enjoyable aspect of the role:

“Just seeing people go from not understanding to understanding a topic because of my help”

Similar themes were identified in stage 2, with an additional role described: that of clarifying misconceptions from learners who professed to be somewhat knowledgeable about the subject:

“Presumed knowledge was a little dangerous as partially informed learners were posting well-intentioned but incorrect information in threads that needed intervention for clarification. They posted these (comments) with presumed authority which mislead the learners at times” (Mentor 8, HSST)

When presented with a series of statements in the stage 1 survey about potential challenges, the responses show that none of these were commonly encountered by the nine respondents. However, another challenge was raised which related to the level and depth in which to respond to a question.

“There was always a bit of a worry that my answers to questions would not be at the right level for the learner. Either too complex or too simple and therefore potentially patronizing” (Survey Responce)

#### Mentors Found Mentoring an Unexpected Learning Opportunity

The findings from both stages 1 and 2 showed that many of the mentors had, on reflection, understood more about how different people view genomics and were more understanding of the patient perspective after their mentoring experience. Some mentors also identified new skills they had developed as a result of mentoring.

Most mentors are involved in training junior colleagues as part of their every-day role. Many mentioned how they would take the skills that they had learnt through this experience and apply them in their own practice.

*“The process of being a mentor itself allowed me to reflect on how I can improve my own training skills—ways of using open questions to stimulate independent thought” (Mentor 10, HSST)*

Mentors also reflected on how they learnt from the learners. Many mentors mentioned how this experience increased their awareness of the diversity of views about genomics.

*“I really enjoyed this and liked how it promoted questions and ideas of my own as a result of seeing such a wide range of posts made by many different people. I felt like I was educated too!” (Survey Responce)*

“… (to see) the different types of discussions from different users and different backgrounds. I think it is quite eye opening ” (Mentor 1, HSST)

Another common theme was the value that patients (and their families) brought to the course. Like the comments posted by learners, comments from patients enhanced the mentors’ experiences.

“They really helped me to reflect on the significance of my own work, so it was interesting in putting the whole field into perspective, a different perspective. I guess it’s a bit like sitting in a clinic, how people went through it, questions they have, the uncertainties and the fear, anxieties, the whole human dimension.” (Mentor 2, HSST)

Given feedback from learners, mentors also found themselves reflecting on perceptions of their own professional role:

“Realizing I’m part of a group of genomics professionals involved in work that other health professionals and the public/patients view in wonderment and amazement.” (Survey Responce)

“Genomics is not just in my office it’s everywhere, people are interested.” (Mentor 2, HSST)

## Discussion

Our course ’Whole Genome Sequencing: Decoding the Language of Life and Health’ was well-received by learners, including healthcare professionals. While recognizing the biased sample of the learners who provided comments and completed the post-course survey, those that did complete the final reflection step felt they had an improved understanding of WGS and greater awareness of the current applications and limitations of this technology in healthcare after completing the course.

The FutureLearn platform, and course structure, was well-received by learners. Preferred features included the availability of content in different formats, the flexible nature of the course, learning as a cohort and social learning. The FutureLearn platform encourages learners to engage with the course and expand the discussion by drawing on individuals’ different perspectives (Sharples et al., 2014), and is built on the foundations of Conversation Theory (Pask, 1976) and Conversational Framework (Laurillard, 2002). Central to this framework is the continual dialogue between learner and teacher (or in our case, mentor), as well as between learners, which extends the learning experience. While FutureLearn encourages the peer-to-peer learning model, the results from our evaluation demonstrate the critical role mentors play in overseeing these conversations to ensure any misconceptions raised by well-intentioned learners are not perpetuated as ’scientific fact’.

### The Mentoring Model Can Act as a Template for Other MOOCS

One of the most successful features from the learners’ perspective was the mentors. The model we used, based on Leon Urrutia et al. (2015), ensured mentors were well prepared for the role, understood the expected duties and responsibilities, and were supported throughout their experience. This validates the system reported by Leon Urrutia et al. (2015) demonstrating the effectiveness of establishing a virtual reporting system in order to ensure a connected mentoring team. We have used the mentors’ evaluation data to refine the induction sessions (as shown in **Figure 2**), and to monitor mentors’ activities in subsequent runs. Sustainability of mentoring MOOCs has been raised in the literature, with studies identifying work-related implications for mentors due to unrealistic expectations of workload (Sinclair et al., 2015, Leon Urrutia et al., 2016, Watolla, 2016). This was not an issue raised in our evaluation, likely due to two factors: the use of rotas to organize mentor shifts to fit in with their day-to-day workload; and the fact that mentors could use their activity as evidence for their training program portfolios. In addition, our model is potentially more sustainable since it draws on mentors from large populations of trainees rather than a much smaller academic team (Leon Urrutia et al., 2016, Watolla, 2016).

This evaluation also highlighted unexpected benefits for the mentors. Some of these benefits, such as learning new skills that can be applied in their own training practice and appreciating the diversity of views about the topic area, have previously been reported by Leon Urrutia et al. (2016) who explored the experiences of PhD students as MOOC mentors. Our mentors also identified additional benefits such as gaining a new perspective on their own role in the clinical pathway and hearing from patients about the impact of genomic testing. Although not patient facing, as they are based in clinical laboratories, acting as a mentor has provided them with an opportunity to interact with and hear from patients firsthand, which they do not usually experience as part of their day-to-day practice. Additionally, this experience could also be used as evidence of patient interaction for their training program (The Royal College of Pathologists, 2015, National School of Healthcare Science, 2016).

### Taking This Work Forward

The need to support NHS staff in education, training and professional development in all specialties has been highlighted in recent NHS policy documents (NHS England, 2019b, NHS England, 2019a). Investment in continuing professional development (CPD) for NHS staff has decreased over recent years, and there has been a recent call for this to be reversed (NHS England, 2019a). The findings from this study suggest using mentors to facilitate MOOCs may be one avenue to explore as a sustainable approach to the provision of high-quality healthcare CPD opportunities, especially where scale is required. Although the student cohort who undertake MOOCs can be quite diverse, the learning can be personalized, as mentors can intervene and elevate the learning by engaging in discussion and signposting to additional resources (Leon Urrutia et al., 2015).The investment required for the development and sustainability of MOOCs could be offset by savings from the costs of releasing staff to attend face-to-face events.

We have shown this model of learning is acceptable to healthcare staff with the added benefit of providing professional development for mentors. During more recent runs of this MOOC recruitment of mentors has expanded to other genomic professions (genetic counsellors, bioinformaticians) with similar benefits seen (unpublished data). As genomic medicine becomes embedded in mainstream care recruitment could be upscaled to include healthcare professionals not typically associated with genomics. Just like other online courses, MOOCs can be used as stand-alone educational resource, as seen here, or part of a structured course (Yousef et al., 2014, Cornelius et al., 2019). As with all educational material, investment will still be required to support the up-front-costs of course development, and mechanisms will need to be in place to keep course content current. While we have shown the benefit of using frontline healthcare professionals as mentors for our MOOC, more research will be needed to see if this mentor model can be replicated for other healthcare professional groups and in other healthcare settings.

### Conclusion

MOOCs are an excellent vehicle for reaching large numbers of learners from across healthcare professions. The use of frontline practitioners as course mentors was successful in this setting: these mentors enhanced the learning experience, while the model itself developed frontline staff as educators. Further research is needed to see if this model, which may offer a sustainable way to deliver healthcare MOOCs, can be replicated, both in terms of using different professional groups as mentors and in healthcare settings outside of the NHS.

## Data Availability Statement

The datasets generated for this study are available on request to the corresponding author.

## Ethics Statement

Ethical review and approval was not required for the study on human participants in accordance with the local legislation and institutional requirements. The patients/participants provided their written informed consent to participate in this study.

## Author Contributions

MB conceived the idea for publication. MB and StS had intellectual input into the evaluation design. MB, SS, and StS contributed to data collection and analysis. MB, EM, AM, SS, StS, and AS provided intellectual input into preparation of the manuscript. All authors approved the final version and agree to be accountable for all aspects of the work.

## Funding

This work was supported by Health Education England’s Genomics Education Program.

## Conflict of Interest

The authors declare that the research was conducted in the absence of any commercial or financial relationships that could be construed as a potential conflict of interest.
